# General health status in Iranian diabetic patients assessed by short-form-36 questionnaire: a systematic review and meta-analysis

**DOI:** 10.1186/s12902-018-0262-2

**Published:** 2018-05-31

**Authors:** Masoud Behzadifar, Rahim Sohrabi, Roghayeh Mohammadibakhsh, Morteza Salemi, Sharare Taheri Moghadam, Masood Taheri Mirghaedm, Meysam Behzadifar, Hamid Reza Baradaran, Nicola Luigi Bragazzi

**Affiliations:** 1 Social Determinants of Health Research Center, Lorestan University of Medical Sciences, Khorramabad, Iran; 2 Iranian Social Security Organization, Zanjan Province Health Administration, Zanjan, Iran; 3 Department of Health Services Management, School of Health Management and Information Sciences, Iran University of Medical Sciences, Tehran, Iran; 4 Social Determinants in Health Promotion Research Center, Hormozgan University of Medical Sciences, Bandar Abbas, Iran; 5 Health Management and Economics Research Center, Iran University of Medical Sciences, Tehran, Iran; 6 Endocrine Research Center Institute of Endocrinology and Metabolism, Iran University of Medical Sciences, Tehran, Iran; 7 School of Public Health, Department of Health Sciences (DISSAL), University of Genoa, Genoa, Italy

**Keywords:** General health status, Diabetes, Short-Form-36 questionnaire, Iran, Meta-analysis

## Abstract

**Background:**

Diabetes mellitus is one of the most prevalent diseases worldwide. Diabetes is a chronic disease associated with micro- and macro-vascular complications and deterioration in general health status. Therefore, the aim of this study was to estimate general health status among Iranian diabetic patients through a systematic review and meta-analysis of study utilizing the Short-Form-36 questionnaire.

**Methods:**

Searching the EMBASE, PubMed, ISI/Web of Sciences (WOS), MEDLINE via Ovid, PsycoINFO, as well as Iranian databases (MagIran, Iranmedex, and SID) from January 2000 to December 2017. The methodological quality of the studies was evaluated using the “A Cochrane Risk of Bias Assessment Tool: for Non-Randomized Studies of Interventions” (ACROBAT-NRSI). Random-effect model was used and the means were reported with their 95% confidence interval (CI). To evaluate the heterogeneity between studies, I^2^ test was used. Egger’s regression test was used to assess the publication bias.

**Results:**

Fourteen studies were retained in the final analysis. The mean general health status using SF-36 in diabetic patients of Iran was 51.9 (95% CI: 48.64 to 53.54). The mean physical component summary was 52.92 [95% CI: 49.46–56.38], while the mean mental component summary was 51.02 [95% CI: 46.87–55.16].

**Conclusion:**

The findings of this study showed that general health status in Iranian diabetic patients is low. Health policymakers should work to improve the health status in these patients and take appropriate interventions.

**Electronic supplementary material:**

The online version of this article (10.1186/s12902-018-0262-2) contains supplementary material, which is available to authorized users.

## Background

Diabetes mellitus is one of the most prevalent diseases worldwide, imposing a relevant epidemiological and clinical burden, both in terms of deaths and morbidities. The prevalence of diabetes is increasing both in developed and developing countries, and has doubled over the past three decades, with almost 80% of diabetic patients living in less developed countries [1, 2]. Population aging, lifestyle changes, lack of mobility, and many other factors characterizing modern life have contributed to such an increase [3]. In 2014, the prevalence of diabetes in people aged greater than 18 years in the world was about 8.5%. It is anticipated that diabetes will be the seventh cause of death by 2030, and, despite all efforts to control the disease, it still remains one of the major public health challenges [4]. The number of people with diabetes is expected to rise up to about 592 million by 2035 [5]. The prevalence of diabetes in the Middle East and North Africa is about 10.9%. In these areas, about 35 million people are affected by diabetes, with Iran having the highest prevalence (9.94%) among the countries of the Middle East [6].

Such concerns necessitate adequate health policies in order to control and prevent diabetes [7]. This disorder represents a chronic disease associated with micro- and macro-vascular complications, which dramatically impact on general health status [8]. Studies have shown that such complications can affect physical, mental and social life of people, modifying and interfering with their usual every day functioning [9]. Hence, treatments of diabetes are usually evaluated based on their effect on health status [10], which, as a key factor in effectiveness studies, refers, indeed, to the mental, physical and social status of the patient [11]. Considering the general health status among diabetic patients can provide care givers with a better understanding of patients’ conditions, indicating which health provisions are necessary for a proper management of the disease [12].

To assess general health status among diabetes patients, a variety of questionnaires have been developed that can measure different dimensions of the patients’ life. The Short-Form 36 (SF-36) questionnaire is one of the most commonly used instruments [13]. It includes 36 questions distributed across eight domains (namely, vitality, physical function, body pain, health perception, physical role, emotional role, social role and mental health) [14, 15].

Various studies have been conducted to assess Iranian diabetic population’s quality of life. Such information can be helpful for measuring the severity of complications and designing and implementing appropriate healthcare policies. In 2013, a review study was conducted in Iran on health status in diabetic patients. In this study, the assessment of health status of diabetics was based on all questionnaires used in Iran. Authors suggested that a meta-analysis study could better provide information about health status in diabetic patients [16]. Therefore, the aim of this study was to estimate general health status among Iranian diabetic patients through a systematic review and meta-analysis of studies utilizing a specific instrument, namely the SF-36 questionnaire.

## Methods

### Literature search

The current study has been performed according to the “The Meta-analysis of Observational Studies in Epidemiology” (MOOSE) guidelines [17]. (Additional file 1).

Two authors independently searched different scholarly electronic databases: namely, EMBASE, PubMed, ISI/Web of Sciences (WOS), MEDLINE via Ovid, PsycoINFO, as well as Iranian databases (MagIran, Iranmedex, and SID). These databases were systematically searched from January 2000 to December 2017 using the following search strategies: (“general health status”) AND (“Short form 36” OR “SF-36” OR “SF-36 health survey questionnaire” OR “Short form-36 health survey questionnaire”) AND (“Diabetes” OR “Diabetic”) AND “Iran”. Studies were searched both in English and Persian (no language filter applied). Reference lists of each included study were also scanned and hand-searched for possible related studies.

#### Inclusion/ criteria

Studies with the following criteria were included if: i) utilizing the SF-36 questionnaire for investigating general health status among Iranian populations, ii) reporting an average score for the eight domains of the questionnaire, iii) reporting both Physical Component Summary (PCS) and Mental Components Summary (MCS) indicators, and iv) reporting means with standard errors (SE) or standard deviations (SD). Both cross-sectional or case-control studies were considered.

#### Exclusion criteria

Studies were excluded if: i) designed as reviews, letters to the editor, editorials, expert opinions, commentaries, clinical trials, case-reports, case-series, or ii) not reporting quantitative details of the SF-36 questionnaire.

#### Quality assessment

The methodological quality of the studies was evaluated using the “A Cochrane Risk of Bias Assessment Tool: for Non-Randomized Studies of Interventions” (ACROBAT-NRSI) [18].

#### Data extraction

Two authors (MB and NLB) extracted the data from the studies, and if there was a controversy between them, another author (AA) resolved the issue. The name of first authors of the studies, the year of publication, the place where the studies were conducted, the number of participants, the duration of diabetes, the design, and the mean scores of SF-36 domains were extracted.

#### Statistical analysis

The pooled value of the mean of overall scores, as well the scores of the eight domains of the questionnaire and the PCS and MCS scores were calculated as the mean and SE. Random-effect model was used and the means were reported with their 95% confidence interval (CI). To evaluate the heterogeneity among studies, I^2^ test was used [19]. For evaluating the potential sources of heterogeneity, subgroup analyses based on the study design, sample size and type of diabetes (type 1 and type 2 diabetes) were conducted. Sensitivity analysis was performed to ensure that the results were stable. This analysis was also performed based on the year of publication. Egger’s regression test was used to assess the publication bias [20].

Finally, case-control studies were pooled together, computing the standardized mean difference (SMD).

Figures with a *p*-value < 0.05 were considered statistically significant. All data were analyzed using Stata 12.0 software (Stata Corp LP, College Station, TX).

## Results

**Fig. 1:**
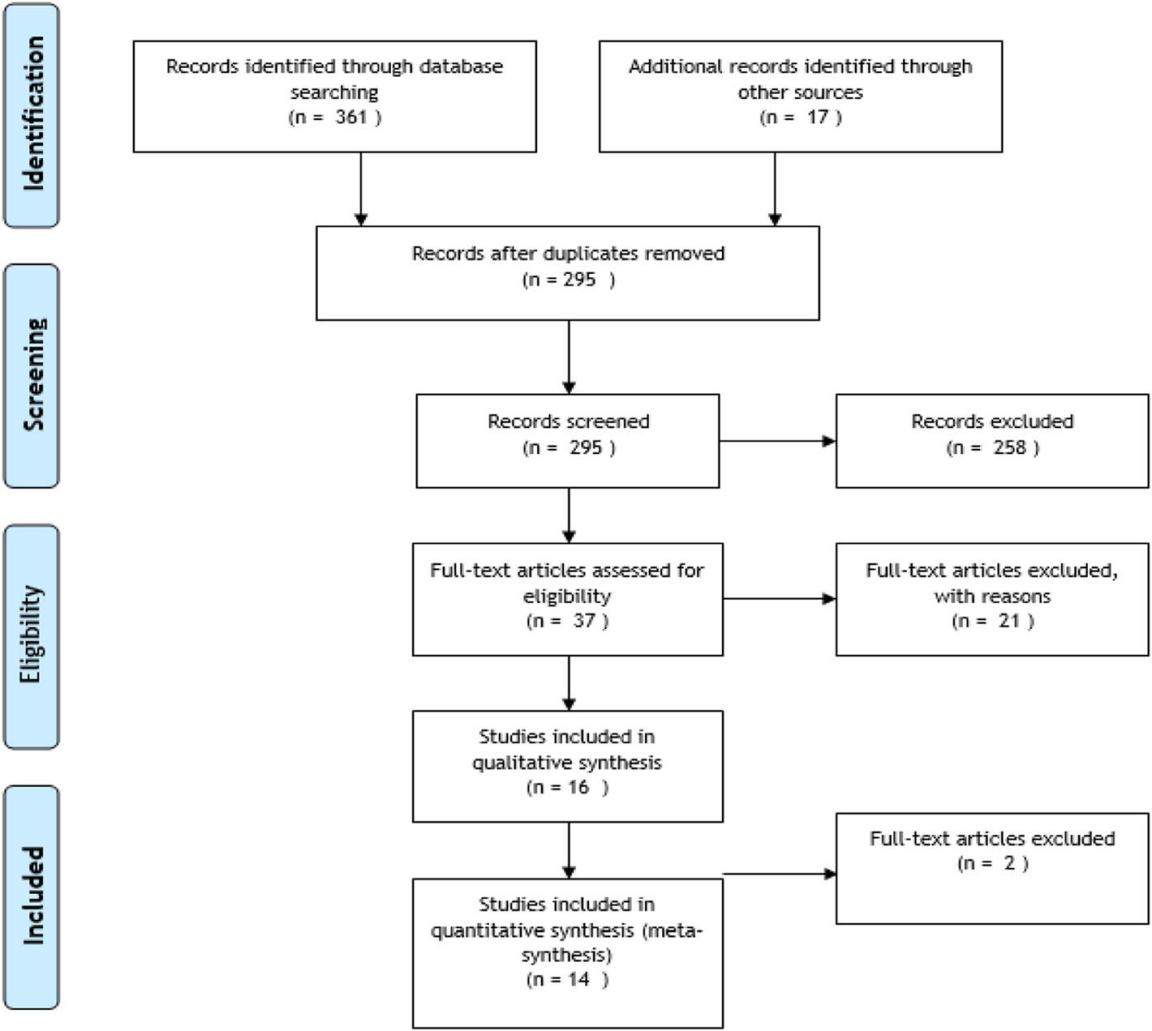
Flowchart of the study retrieval and selection

After the initial electronic database search, 378 studies were found. Eighty-three duplicate studies were deleted. The titles of the retrieved studies were reviewed and 258 studies were excluded due to lack of relevance to the topic. Then, the title and abstract of 37 remaining studies were reviewed by two authors independently and 21 studies were excluded with reason. Finally, the full texts of the remaining 16 studies were examined and, based on the inclusion/exclusion criteria, 14 studies were retained in the final analysis [21–34]. Figure 1 summarizes the stages of the retrieval and selection of the studies.

The included studies were conducted between 2011 and 2017. The total number of participants in the studies was 4492, ranging from 60 to 1847 people. The study designs varied across studies and were cross-sectional for 10 studies and case-control for 4 studies). Table 1 shows the main characteristics of the studies retained in the present systematic review and meta-analysis.

**Table 1:** The main characteristics of the included studies about general health status in Iranian patients with diabetes

First author	Year of publication	Mean score of general health status	Sample size	Female	Male	Age (Mean ± SD)	Type of diabetes	Design of study	Duration of diabetes (Year ± SD)	Married (%)	Setting (City)	Setting (Province)
Borzou	2011	55.53	165	111	54	NA	Type 2	Cross- Sectional	NA	NA	Hamedan	Hamedan
Khaledi	2011	45.23	198	166	32	NA	Type 2	Cross- Sectional	1–5	80.8	Sannadaj	Kurdistan
Saadatjoo	2012	28.52	100	54	46	42.82 ± 16.57	Type 2	Case-Control	NA	82	Birjand	South Khorasan
Timareh	2012	52.97	350	204	146	52.91 ± 11.7	Both type	Cross- Sectional	NA	86.9	Kermanshah	Kermanshah
Sadabadi	2013	44.72	60	NA	NA	NA	Type 2	Case-Control	NA	NA	Tabriz	East Azerbaijan
Darvishpoor Kakhki	2013	46.2	131	79	52	NA	Type 2	Case-Control	NA	80.2	Tehran	Tehran
Hadi	2013	54.11	300	222	78	50.98	Both type	Cross- Sectional	NA	84	Shiraz	Fars
Darvishpoor Kakhki	2013	52.11	140	NA	NA	47.3 ± 12.7	Both type	Cross- Sectional	8.83 ± 6.10	NA	Tehran	Tehran
Mohammadshahi	2015	51.81	110	51	59	53.4 ± 8.12	Type 2	Cross- Sectional	NA	NA	Ahvaz	Khuzestan
Kashfi	2015	61.33	124	89	35	59.65 ± 12.3	Type 2	Case-Control	7.68 ± 6.93	83.9	Larestan	Fars
Borhaninejad	2016	46.48	120	69	51	71.32 ± 5.13	Type 2	Cross- Sectional	NA	73.4	Kerman	Kerman
Hajian-Tailaki	2016	56.27	747	372	375	68 ± 7.6 in male and 67.7 ± 7.9 in female	Type 2	Cross- Sectional	NA	NA	Babol	Mazandaran
Mazloomy Mahmood Abad	2017	59.27	100	59	41	51.92 ± 11.53	Type 2	Cross- Sectional	NA	94	Sirjan	Kerman
Gholami	2017	51.11	1847	1289	558	59.65 ± 12.3	Type 2	Cross- Sectional	NA	19.9	Nishabur	Razavi Khorasan

**Fig. 2:**
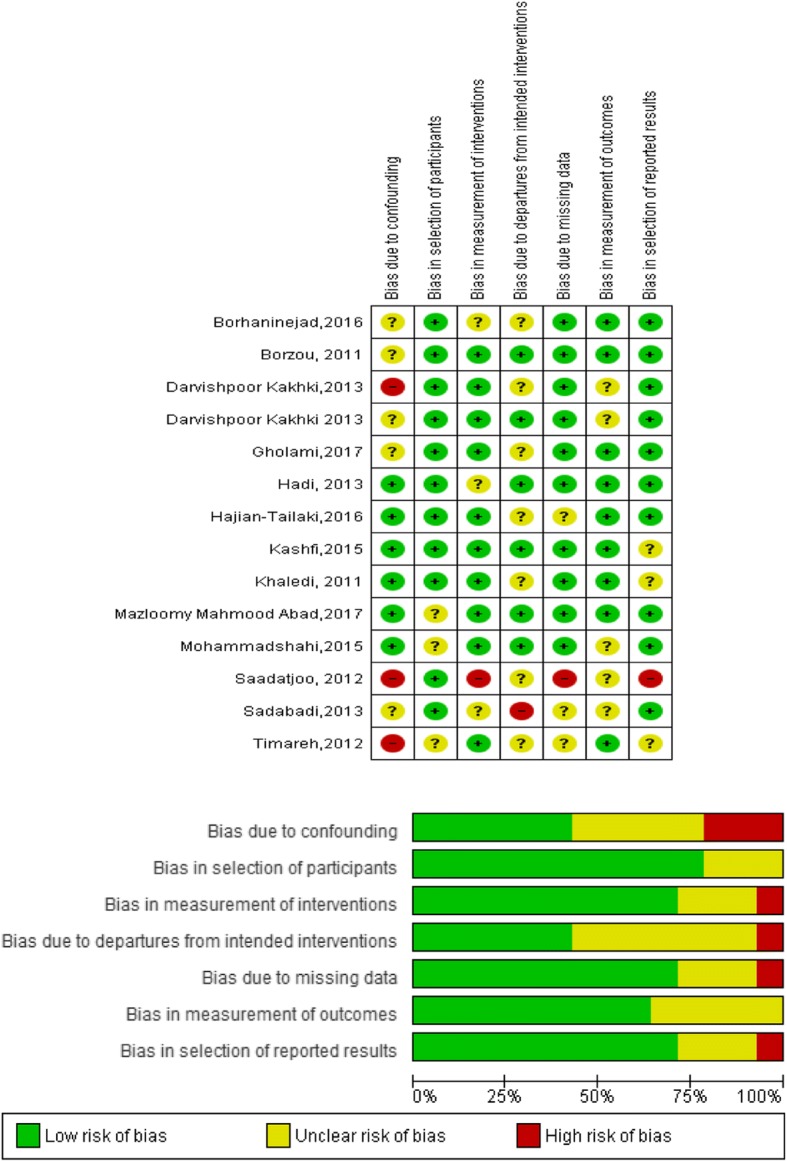
The result of quality assessment of risk of bias of included studies

The quality assessment of the risk of bias of the included studies is shown in Table 2 and Fig. 2.

**Table 2:** Risk of Bias Assessment of included studies based on the ACROBAT-NRSI instrument

Study	Domains of bias						
	Bias due to confounding	Bias in selection of participants	Bias in measurement of interventions	Bias due to departures from intended interventions	Bias due to missing data	Bias in measurement of outcomes	Bias in selection of reported results
Borzou	Moderate risk	Low risk	Low risk	Low risk	Low risk	Low risk	Low risk
Khaledi	Low risk	Low risk	Low risk	Moderate risk	Low risk	Low risk	Moderate risk
Saadatjoo	Serious risk	Low risk	Serious risk	Moderate risk	Serious risk	Moderate risk	Serious risk
Timareh	Serious risk	Moderate risk	Low risk	Moderate risk	Moderate risk	Low risk	Moderate risk
Sadabadi	Moderate risk	Low risk	Moderate risk	Serious risk	Moderate risk	Moderate risk	Low risk
Darvishpoor Kakhki	Serious risk	Low risk	Low risk	Moderate risk	Low risk	Moderate risk	Low risk
Hadi	Low risk	Low risk	Moderate risk	Low risk	Low risk	Low risk	Low risk
Darvishpoor Kakhki	Moderate risk	Low risk	Low risk	Low risk	Low risk	Moderate risk	Low risk
Mohammadshahi	Low risk	Moderate risk	Low risk	Low risk	Low risk	Moderate risk	Low risk
Kashfi	Low risk	Low risk	Low risk	Low risk	Low risk	Low risk	Moderate risk
Borhaninejad	Moderate risk	Low risk	Moderate risk	Moderate risk	Low risk	Low risk	Low risk
Hajian-Tailaki	Low risk	Low risk	Low risk	Moderate risk	Moderate risk	Low risk	Low risk
Mazloomy Mahmood Abad	Low risk	Moderate risk	Low risk	Low risk	Low risk	Low risk	Low risk
Gholami	Moderate risk	Low risk	Low risk	Moderate risk	Low risk	Low risk	Low risk

**Fig. 3:**
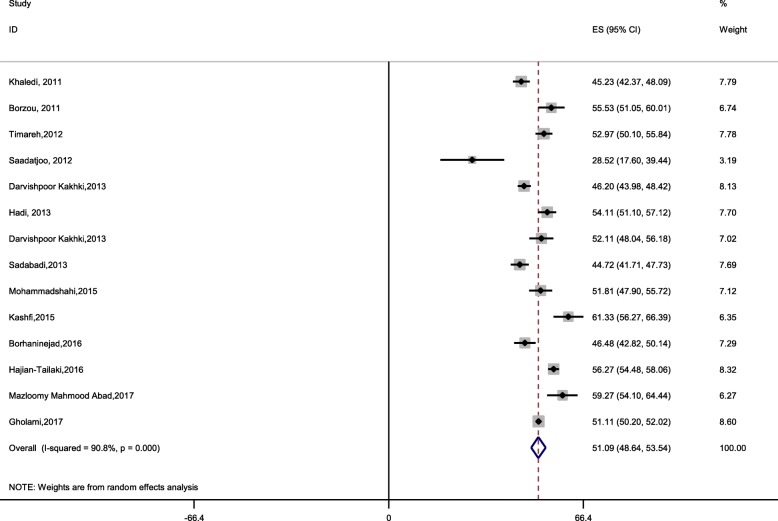
The Mean health status in Iranian diabetic patients (2011–2017), based on the random-effects model

The mean general health status using SF-36 based on the random-effect model in diabetic patients of Iran was 51.9 (95% CI: 48.64 to 53.54). The lowest health status was observed in the study of Saadatjoo with a score of 28.52 and the highest in Kashifi’s study, with a value of 61.33. Figure 3 shows the overall general health status among the included studies.

**Fig. 4:**
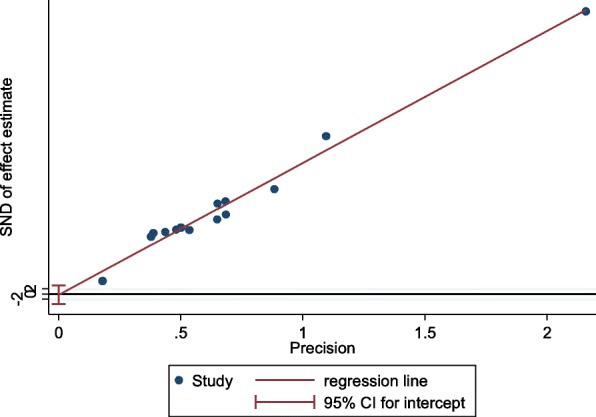
Probability of publication bias in the included studies

Using the Egger’s test, no publication bias could be detected (*p* = 0.859, see Fig. 4).

To investigate the possible sources of heterogeneity between studies, subgroup analysis was conducted based on study design, sample size and study quality. Table 3 shows the results of subgroup analysis.

**Table 3:** The results of subgroup analysis

Variables	Number of studies	Number of participants	Mean score of general health status (95% CI)	I^2^	*P*-value
Design of studies					
Cross-sectional	10	4077	52.32 (50.02–54.62)	86.8%	0.001
Case-control	4	415	46.47 (38.87–54.08)	93.3%	0.001
Sample size					
≤120	6	614	49.58 (43.11–56.05)	92%	0.001
> 120	8	3878	51.60 (48.95–54.24)	90.7%	0.001
Type of diabetes					
Type 2	11	3702	50.46 (47.43–53.49)	92.46%	0.001
Both type (type 1 and 2)	3	790	53.22 (51.37–55.07)	0%	0.001

For further evaluation of sources of heterogeneity, the results of meta-regression were analyzed based on the year of publication and the sample size of studies, as presented in Table 4. The results showed that the quality of life of diabetic patients has increased on a yearly basis and has decreased based on the sample size. However, none of the results were statistically significant.

**Table 4:** The results of meta-regression

Variables	Coefficient	S.E.	t	*P*-value	Lower 95%	Upper 95%
Year	1.36	1.07	1.27	0.22	−0.99	3.73
Sample size	−0.00	0.00	−0.22	0.82	−0.01	0.00

**Fig. 5:**
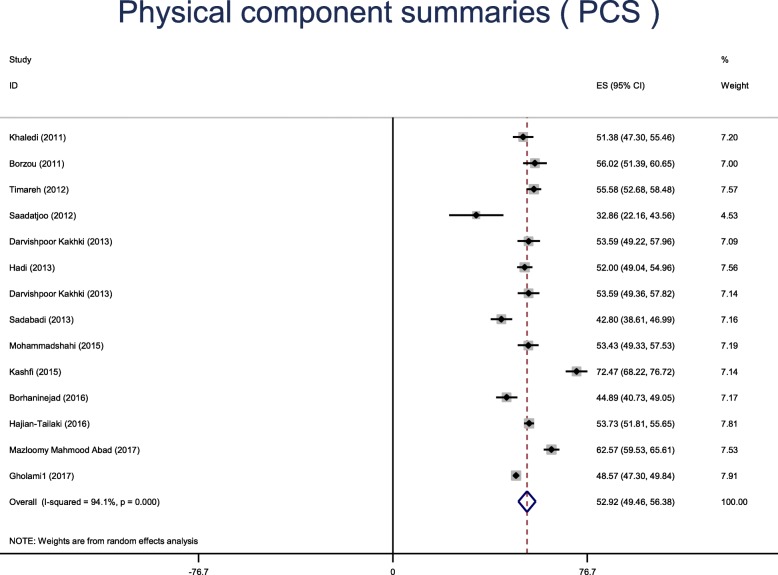
The Physical component summaries (PCS)

**Fig. 6:**
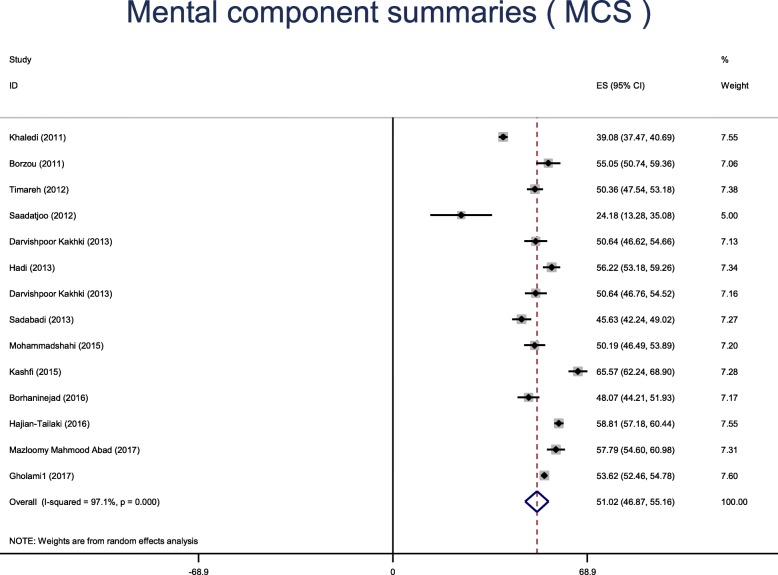
The mental component summaries (MCS)

The results based on the eight domains of the SF-36 questionnaire are presented in Table 5. The mean scores of PCS and MCS are shown in Figs. 5 and 6**.** The mean of PCS was 52.92 [95% CI: 49.46–56.38], while the mean of MCS was 51.02 [95% CI: 46.87–55.16].

**Table 5:** The health status based on the 8 domains of the SF-36 questionnaire

Variables	Mean (95% CI)	Heterogeneity		*P*-value of publication bias
		I^2^	*P*-value	
Physical function	61.62 (55.70–67.53)	98.6%	0.001	0.78
Role physical	49.96 (44.50–55.41)	95.6%	0.001	0.83
Body pain	52.26 (48.47–56.04)	95.8%	0.001	0.57
General health	47.34 (44.15–50.53)	96.5%	0.001	0.01
Vitality	46.99 (43.28–50.69)	97.4%	0.001	0.64
Social function	57.86 (46.87–68.85)	99.7%	0.001	0.15
Role emotional	50.38 (45.29–55.47)	97.4%	0.001	0.28
Mental health	47.79 (40.06–55.52)	99.6%	0.001	0.32

**Fig. 7:**
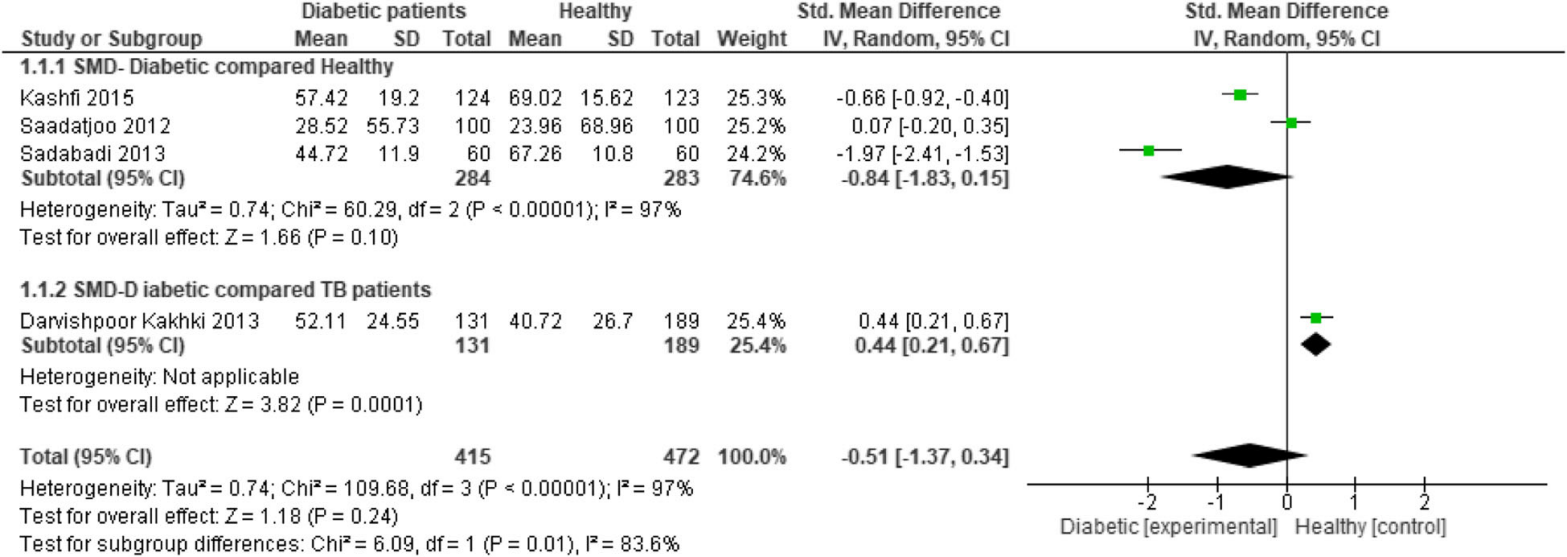
The results of pooling together case-control studies

Finally, case-control studies were pooled together (Fig. 7). The general health status of diabetic patients compared to healthy controls was lower with a SMD of − 0.84 [95% CI: -1.83 to 0.51] and compared to the group of patients with tuberculosis with a SMD of 0.44 [95% CI: 0.21- 0.67].

## Discussion

In the 14 studies included in this systematic review and meta-analysis, numerous complications and co-morbidities were reported in people with diabetes. Health policy- and decision-makers should pay attention to the implications of the reduced general health status in diabetic patients in Iran. Various studies have, indeed, shown that health status is an independent prognostic predictor of survival and hospitalization rate in patients with peripheral arterial and renal patients, and of mortality in patients with coronary heart disease [35–38].

General health status is decreased in diabetic patients [39], when compared to the health status of general population, which, in a recent study, reported an average score of 67.69 ± 14.78 [40]. Healthcare providers should be aware of the patients’ perspective and their perceived health. Preventing further diabetes complications and providing better conditions for patients’ lives is fundamental. Physical and mental interventions can improve the health status of diabetic patients and avoid, or at least delay, further deterioration [41].

Our findings showed that the dimensions of physical and social function had the highest score whereas the lowest score was related to vitality and general health. The results of our study are consistent with the study done in Brazil [42], whereas other studies reported higher values [43–45]. The level of access to health services, the economic and social conditions of people, the physical and mental conditions of individuals can, at least partially, explain these differences [46, 47]. Some studies point to the existence of health inequalities in that people with a higher socioeconomic status have more incentive and energy to change their livelihood and are more involved in their own health care processes [48]. An important cross-sectional survey of 13 national samples from Asia, Australia, Europe and North America of 5104 patients with diabetes from the multinational study of Diabetes Attitudes, Wishes and Needs (DAWN) has shown that the reported levels of well-being, self-management, and diabetes control correlate with country, respondent demographic and disease characteristics, as well as with healthcare features [49]. These findings have been replicated by a follow-up study [50].

The findings of the present study indicate that diabetes dramatically affects vitality and general health domains; hence these areas should be given more attention when treating diabetic patients. In our study, MCS was less than PCS, which was consistent with the results of the Al-Shehri study [51]. Various studies have been conducted to show that mental disorders such as depression in patients with diabetes can be remarkably observed. In a review, results showed that depression in diabetic patients had a negative effect on the treatment process and increased complications of the disease [52].

It seems that the chronic and severe nature of diabetes mellitus in the long run leads to a decrease in the general health status [53]. It should be noted that the core of the concept of reported/perceived health status is a feeling/perception of one’s own health and, in fact, other aspects of the health status form a sense of health that is low in patients with diabetes. Affecting the emotional aspects impacts on energy and vitality of patients with diabetes. Other studies have also shown a decrease in vitality, with an increase of fatigue, depression, anxiety and stress problems, among patients with diabetes. Therefore, diabetes has a long-term negative effect on the health of patients. The decrease in the health status in patients with diabetes has also been replicated in other studies [54].

These observations can be confirmed if we compare health status of Iranian subjects with diabetes with the health status of people with chronic-degenerative disorders, such as rheumatoid arthritis with an average score of 52.47 [55], or cardiovascular disorders with a mean of 53.19 [56], among others. Similarly, low scores have been found for asthma [57] or chronic kidney disease [58]. Scores even lower (40.43 ± 12.7) were reported for individuals with drug addiction [59].

In meta-analysis studies, taking into account potential sources of heterogeneity is crucial [60]. To investigate this aspect, we performed subgroup-analysis based on each SF-36 scale domain. The results of meta-regression were also studied for further evaluation of heterogeneity sources, which showed an increased average health status of diabetic patients based on the year of publication, even though not statistically significant. In recent years the status of services provided to diabetics is on the rise, but it seems that many of the services provided to them are not of sufficient standards, and the quality of care for these patients should be monitored more closely by healthcare providers in Iran.

However, this study has some limitations that should be properly mentioned. First, the primary studies missed to give some complementary information about patients, such as sex, other illnesses/co-morbidities, education level and income. Second, a high level of heterogeneity was observed, which can be attributed to methodological differences. Third, the health status in diabetic patients has not been studied in many Iranian provinces, which can challenge the generalizability of our estimation to all Iranian diabetic population.

## Conclusion

The findings of this study showed that general health status in Iranian diabetic patients is low. Health policy- and decision-makers should work to improve the health status in these patients and take appropriate interventions. Therefore, it is recommended to look at important factors such as patients’ attitudes in changing and improving their lifestyle. A combination of both clinical and non-clinical interventions should be targeted at increasing the standard of living of these patients.

## Additional file

Additional file 1: MOOSE Guidelines for Meta-Analyses and Systematic Reviews of Observational Studies. (DOCX 22 kb)

## Abbreviations

ACROBAT-NRSI: A Cochrane Risk of Bias Assessment Tool: for Non-Randomized Studies of Interventions
BP: Bodily pain
CI: Confidence intervals
ERF: Emotional role functioning
GHP: General health perceptions
MCS: Mental Component Summary
MeSH: Medical Subject Headings
MH: Mental health
PCS: Physical Component Summary
PF: Physical functioning
PRF: Physical role functioning
PROs: Patient-reported outcomes
SF-36: The Short-Form 36
SID: Scientific Information Database
SMD: Standardized Mean Difference
SRF: Social role functioning
VT: Vitality
WOS: Web of Science

## References

[1] Zimmet P, Alberti KG, Magliano DJ, Bennett PH (2016). Diabetes mellitus statistics on prevalence and mortality: facts and fallacies. Nat Rev Endocrinol.

[2] NCD Risk Factor Collaboration (NCD-RisC) (2016). Worldwide trends in diabetes since 1980: a pooled analysis of 751 population-based studies with 4.4 million participants. Lancet.

[3] Speight J, Reaney MD, Barnard KD (2009). Not all roads lead to Rome-a review of quality of life measurement in adults with diabetes. Diabet Med.

[4] Mathers CD, Loncar D (2006). Projections of global mortality and burden of disease from 2002 to 2030. PLoS Med.

[5] Guariguata L, Whiting DR, Hambleton I, Beagley J, Linnenkamp U, Shaw JE (2014). Global estimates of diabetes prevalence for 2013 and projections for 2035. Diabetes Res Clin Pract.

[6] Javanbakht M, Mashayekhi A, Baradaran HR, Haghdoost A, Afshin A (2015). Projection of diabetes population size and associated economic burden through 2030 in Iran: evidence from micro-simulation Markov model and Bayesian meta-analysis. PLoS One.

[7] Wild S, Roglic G, Green A, Sicree R, King H (2004). Global prevalence of diabetes: estimates for the year 2000 and projections for 2030. Diabetes Care.

[8] Papadopoulos AA, Kontodimopoulos N, Frydas A, Ikonomakis E, Niakas D (2007). Predictors of health-related quality of life in type II diabetic patients in Greece. BMC Public Health.

[9] Colberg SR, Sigal RJ, Fernhall B, Regensteiner JG, Blissmer BJ, Rubin RR (2010). Exercise and type 2 diabetes: the American College of Sports Medicine and the American Diabetes Association: joint position statement. Diabetes Care.

[10] Gusmai Lde F, Novato Tde S, Nogueira Lde S (2015). The influence of quality of life in treatment adherence of diabetic patients: a systematic review. Rev Esc Enferm USP.

[11] American Diabetes Association (2016). Standards of medical care in diabetes-2016 abridged for primary care providers. Clin Diabetes.

[12] International Diabetes Federation Guideline Development Group (2014). Global guideline for type 2 diabetes. Diabetes Res Clin Pract.

[13] Ware JE, Sherbourne CD (1992). The MOS 36-item short-form health survey (SF-36). Med Care.

[14] Al Hayek AA, Robert AA, Al Saeed A, Alzaid AA, Al Sabaan FS (2014). Factors associated with health-related quality of life among Saudi patients with type 2 diabetes mellitus: a cross-sectional survey. Diabetes Metab J.

[15] Lyons RA, Perry HM, Littlepage BN (1994). Evidence for the validity of the short-form 36 questionnaire (SF-36) in an elderly population. Age Ageing.

[16] Kiadaliri AA, Najafi B, Mirmalek-Sani M (2013). Quality of life in people with diabetes: a systematic review of studies in Iran. J Diabetes Metab Disord.

[17] Stroup DF, Berlin JA, Morton SC, Olkin I, Williamson GD, Rennie D (2000). Meta-analysis of observational studies in epidemiology: a proposal for reporting. Meta-analysis of observational studies in epidemiology (MOOSE) group. JAMA.

[18] Sterne JA, Hernán MA, Reeves BC, Savović J, Berkman ND, Viswanathan M, et al. ROBINS-I: a tool for assessing risk of bias in non-randomised studies of interventions. BMJ. 2016;355:i4919. 10.1136/bmj.i4919 PMC5062054 27733354

[19] Higgins JP, Thompson SG, Deeks JJ, Altman DG (2003). Measuring inconsistency in meta-analyses. BMJ.

[20] Egger M, Davey Smith G, Schneider M, Minder C (1997). Bias in meta-analysis detected by a simple, graphical test. BMJ.

[21] Borzou SR, Salavati M, Safari M, Hadadinejad S, Zandieh M, Torkaman B (2011). Quality of life in type II diabetic patients referred to Sina hospital, Hamadan. ZJRMS.

[22] Khaledi S, Moridi G, Gharibi F (2011). Survey of eight dimensions quality of life for patients with diabetes type II, referred to Sanandaj diabetes center in 2009. J Fasa Univ Med Sci.

[23] Saadatjoo S, Rezvanee M, Tabyee S, Oudi D (2012). Life quality comparison in type 2 diabetic patients and none diabetic persons. Mod Care J.

[24] Timareh M, Rhimi M, Abbasi P, Rezaei M, Hyaidarpoor S (2012). Quality of life in diabetic patients referred to the Diabete research Center in Kermanshah. J Kermanshah Univ Med Sci.

[25] Darvishpoor Kakhki A, Abed Saeedi J, Masjedi MR, Askari H (2013). Comparison of life quality of diabetic patients with TB patients. J Knowledge Health.

[26] Darvishpoor Kakhki A, Abed saeedi Z (2013). Health-related quality of life of diabetic patients in Tehran. Int J Endocrinol Metab.

[27] Hadi N, Ghahramani S, Montazeri A (2013). Health related quality of life in both types of diabetes in Shiraz, Iran. Shiraz E-Med J.

[28] Hatamloo Sadabadi M, Babapour KJ (2013). Comparison of Quality of Life and Coping Strategies in Diabetic and Non Diabetic People. JSSU.

[29] Kashfi SM, Nasri A, Dehghan A, Yazdankhah M (2015). Comparison of quality of life of patients with type II diabetes referring to diabetes Association of Larestan with healthy people in 2013. J Neyshabur Univ Med Sci.

[30] Mohammadshahi M, Shirani F, Elahi S, Ghasemi S, Alayi Shahni M, Haidari F (2015). Evaluation of relationship between dietary patterns and quality of life in patients with type 2 diabetes. Daneshvarmed.

[31] Borhaninejad V, Kazazi L, Haghi M, Chehrehnegar N (2016). Quality of life and its related factors among elderly with diabetes. Salmand.

[32] Hajian-Tilaki K, Heidari B, Hajian-Tilaki A (2016). Solitary and combined negative influences of diabetes, obesity and hypertension on health-related quality of life of elderly individuals: a population-based cross-sectional study. Diabetes Metab Syndr.

[33] Gholami A, Khazaee-Pool M, Rezaee N, Amirkalali B, Abbasi Ghahremanlo A, Moradpour F (2017). Household food insecurity is associated with health-related quality of life in rural type 2 diabetic patients. Arch Iran Med.

[34] Mazloomy S, Rezaeian M, Naghibzadeh Tahami A, Sadeghi R (2017). Association between Health–Related Quality of Life and Glycemic Control in Type 2 Diabetics of Sirjan City in 2015. JRUMS.

[35] Issa SM, Hoeks SE, Scholte op Reimer WJ, Van Gestel YR, Lenzen MJ, Verhagen HJ (2010). Health-related quality of life predicts long-term survival in patients with peripheral artery disease. Vasc Med.

[36] Lopes AA, Bragg-Gresham JL, Satayathum S, McCullough K, Pifer T, Goodkin DA (2003). Health-related quality of life and associated outcomes among hemodialysis patients of different ethnicities in the United States: the Dialysis outcomes and practice patterns study (DOPPS). Am J Kidney Dis.

[37] Mommersteeg PM, Denollet J, Spertus JA, Pedersen SS (2009). Health status as a risk factor in cardiovascular disease: a systematic review of current evidence. Am Heart J.

[38] Parkerson GR, Gutman RA (2000). Health-related quality of life predictors of survival and hospital utilization. Health Care Financ Rev.

[39] Schram MT, Baan CA, Pouwer F (2009). Depression and quality of life in patients with diabetes: a systematic review from the European depression in diabetes (EDID) research consortium. Curr Diabetes Rev.

[40] Ghafari R, Rafiei M, Taheri Nejad MR (2014). Assessment of health related quality of life by SF-36 version 2 in general population of Qom city. AMUJ.

[41] Baptista LC, Dias G, Souza NR, Veríssimo MT, Martins RA (2017). Effects of long-term multicomponent exercise on health-related quality of life in older adults with type 2 diabetes: evidence from a cohort study. Qual Life Res.

[42] Nunes-Silva JG, Nunes VS, Schwartz RP, Mlss Trecco S, Evazian D, Correa-Giannella ML (2014). Impact of type 1 diabetes mellitus and celiac disease on nutrition and quality of life. Nutr Diabetes.

[43] Hervás A, Zabaleta A, De Miguel G, Beldarráin O, Díez J (2007). Health related quality of life in patients with diabetes mellitus type 2. An Sist Sanit Navar.

[44] Lindsay G, Inverarity K, McDowell JR (2011). Quality of life in people with type 2 diabetes in relation to deprivation, gender, and age in a new community-based model of care. Nurs Res Pract.

[45] Vázquez VC, González LM, Ruiz EM, Isidoro JM, Ordóñez MS, García CS (2011). Assessment of health outcomes in the type 2 diabetes process. Aten Primaria.

[46] Eljedi A, Mikolajczyk RT, Kraemer A, Laaser U (2006). Health-related quality of life in diabetic patients and controls without diabetes in refugee camps in the Gaza strip: a cross-sectional study. BMC Public Health.

[47] Wubben DP, Porterfield D (2005). Health-related quality of life among North Carolina adults with diabetes mellitus. N C Med J.

[48] De Vogli R, Gimeno D, Kivimaki M (2008). Socioeconomic inequalities in health in 22 European countries. N Engl J Med.

[49] Rubin RR, Peyrot M, Siminerio LM (2006). Health care and patient-reported outcomes: results of the cross-national diabetes attitudes, wishes and needs (DAWN) study. Diabetes Care.

[50] Snoek FJ, Kersch NY, Eldrup E, Harman-Boehm I, Hermanns N, Kokoszka A (2012). Monitoring of individual needs in diabetes (MIND)-2: follow-up data from the cross-national diabetes attitudes, wishes, and needs (DAWN) MIND study. Diabetes Care.

[51] Al-Shehri AH, Taha AZ, Bahnassy AA, Salah M (2008). Health-related quality of life in type 2 diabetic patients. Ann Saudi Med.

[52] Ali S, Stone M, Skinner TC, Robertson N, Davies M, Khunti K (2010). The association between depression and health-related quality of life in people with type 2 diabetes: a systematic literature review. Diabetes Metab Res Rev.

[53] King IM (1994). Quality of life and goal attainment. Nurs Sci Q.

[54] Svenningsson I, Marklund B, Attvall S, Gedda B (2011). Type 2 diabetes: perceptions of quality of life and attitudes towards diabetes from a gender perspective. Scand J Caring Sci.

[55] Karimi S, Yarmohammadian MH, Shokri A, Mottaghi P, Qolipour K, Kordi A (2013). Predictors and effective factors on quality of life among Iranian patients with rheumatoid arthritis. Mater Sociomed.

[56] Yaghoubi A, Tabrizi JS, Mirinazhad MM, Azami S, Naghavi-Behzad M, Ghojazadeh M (2012). Quality of life in cardiovascular patients in Iran and factors affecting it: a systematic review. J Cardiovasc Thorac Res.

[57] Kia NS, Malek F, Ghods E, Fathi M (2017). Health-related quality of life of patients with asthma: a cross-sectional study in Semnan, Islamic Republic of Iran. East Mediterr Health J.

[58] Ghiasi B, Sarokhani D, Dehkordi AH, Sayehmiri K, Heidari MH (2018). Quality of life of patients with chronic kidney disease in Iran: systematic review and meta-analysis. Indian J Palliat Care.

[59] Heidari M, Ghodusi M (2016). Relationship of assess self-esteem and locus of control with quality of life during treatment stages in patients referring to drug addiction rehabilitation centers. Mater Sociomed..

[60] Petitti DB (2001). Approaches to heterogeneity in meta-analysis. Stat Med.

