# Supplementary material for: General health status in Iranian diabetic patients assessed by short-form-36 questionnaire: a systematic review and meta-analysis
Source: BMC Endocr Disord. 2018 May 31;18:34. doi: 10.1186/s12902-018-0262-2 (PMC5984362; doi:10.1186/s12902-018-0262-2)
Supplement: Supplementary file 1 — MOOSE Guidelines for Meta-Analyses and Systematic Reviews of Observational Studies. (DOCX 22 kb) [file 12902_2018_262_MOESM1_ESM.docx]

**Additional file 1: MOOSE Guidelines for Meta-Analyses and Systematic Reviews of Observational Studies**

| **#** | **Section/topic** | **Guideline item** | **Reported on page #** |
| --- | --- | --- | --- |
| **1** | ***Title*** | Identify the study as a meta-analysis (or systematic review) | Title |
|  |  | **Health-related quality of life in Iranian diabetic patients assessed by Short-Form-36 questionnaire: a systematic review and meta-analysis** |  |
| **2** | ***Abstract*** | Use the journal’s structured format | Abstract |
| **3** | ***Introduction*** | The clinical problem | Background |
|  |  | The hypothesis | Background |
|  |  | A statement of objectives that includes the study population, the condition of interest, the exposure or intervention, and the outcome(s) considered | Background |
| **4** | ***Sources*** | Qualifications of searchers (eg, librarians and investigators) | Methods |
|  |  | Search strategy, including time period included in the synthesis and keywords | Methods |
|  |  | Effort to include all available studies, including contact with authors | Methods |
|  |  | Databases and registries searched | Methods |
|  |  | Search software used, name and version, including special features used (eg, explosion) | Methods |
|  |  | Use of hand searching (eg, reference lists of obtained articles) | Methods |
|  |  | List of citations located and those excluded, including justification | Methods |
|  |  | Method of addressing articles published in languages other than English | Methods |
|  |  | Method of handling abstracts and unpublished studies | Methods |
|  |  | Description of any contact with authors | Methods |
| **5** | ***Study Selection*** | Types of study designs considered | Methods |
|  |  | Relevance or appropriateness of studies gathered for assessing the hypothesis to be tested | Methods |
|  |  | Rationale for the selection and coding of data (eg, sound clinical principles or convenience) | Methods |
|  |  | Documentation of how data were classified and coded (eg, multiple raters, blinding, and  interrater reliability) | Methods |
|  |  | Assessment of confounding (eg, comparability of cases and controls in studies where  appropriate) | Methods |
|  |  | Assessment of study quality, including blinding of quality assessors; stratification or  regression on possible predictors of study results | Methods |
|  |  | Assessment of heterogeneity | Methods |
|  |  | Statistical methods (eg, complete description of fixed or random effects models, justification  of whether the chosen models account for predictors of study results, dose-response models,  or cumulative meta-analysis) in sufficient detail to be replicated | Methods |

| **#** | **Section/topic** | **Guideline item** | **Reported on page #** |
| --- | --- | --- | --- |
| **6** | ***Results*** | A graph summarizing individual study estimates and the overall estimate | Results |
|  |  | A table giving descriptive information for each included study | Results |
|  |  | Results of sensitivity testing (eg, subgroup analysis) | Results |
|  |  | Indication of statistical uncertainty of findings | Results |
| **7** | ***Discussion*** | Strengths and weaknesses | Discussion |
|  |  | Potential biases in the review process (eg, publication bias) | Discussion |
|  |  | Justification for exclusion (eg, exclusion of non–English-language citations) | Discussion |
|  |  | Assessment of quality of included studies | Discussion |
|  |  | Consideration of alternative explanations for observed results | Discussion |
|  |  | Generalization of the conclusions (ie, appropriate for the data presented and within the domain  of the literature review) | Discussion |
|  |  | Guidelines for future research | Discussion |
|  |  | Disclosure of funding source | Discussion |

**Reference:** Stroup DF, Berlin JA, Morton SC, Olkin I, Williamson GD, Rennie D, et al. Meta-analysis of observational studies in epidemiology: a proposal for reporting. Meta-analysis of Observational Studies in Epidemiology (MOOSE) group. JAMA. 2000;283(15):2008-12.
